# Discoveries and innovations in cnidarian biology at Cnidofest 2024

**DOI:** 10.1186/s13227-025-00247-5

**Published:** 2025-06-16

**Authors:** Whitney B. Leach, Leslie Babonis, Celina E. Juliano, Nagayasu Nakanishi, Christine E. Schnitzler, Patrick R. H. Steinmetz, Michael J. Layden

**Affiliations:** 1 Department of Biological Sciences, Lehigh University, Bethlehem, PA USA; 2 Department of Ecology and Evolutionary Biology, Cornell University, Ithaca, NY 14853 USA; 3 Department of Molecular and Cellular Biology, University of California, Davis, CA 95616 USA; 4 Fulbright College of Arts and Sciences, Biological Sciences, University of Arkansas, Fayetteville, AR 72701 USA; 5 Whitney Laboratory for Marine Bioscience and Department of Biology, University of Florida, St. Augustine, FL 32080 USA; 6 Michael Sars Centre, University of Bergen, Thormøhlensgt. 55, 5008 Bergen, Norway

**Keywords:** Cnidofest, Cnidarians, *H. vulgaris*, *N. vectensis*, *H. symbiolongicarpus*, Anemone, Polyp, Medusa, Jellyfish

## Abstract

The third iteration of the Cnidarian Model Systems Meeting (Cnidofest) was held August 14–17th, 2024 at Lehigh University in Bethlehem, PA. The meeting featured presentations from laboratories representing 11 countries, covering a broad range of topics related to cnidarian species. The research highlighted diverse topics, with sessions focused on regeneration, evo-devo, genomics, symbiosis, cell biology, physiology, neurobiology, and development. A notable shift at this meeting was the extent to which established cnidarian model systems have caught up with the classical laboratory models such as *Drosophila* and vertebrates, with modern genomic, genetic, and molecular tools now routinely applied. In addition, more cnidarian systems are now being developed for functional studies by the community, enhancing our ability to gain fundamental insights into animal biology that are otherwise difficult in the complex bilaterian model systems. Together, the integration of cnidarian and bilaterian model systems provides researchers with a broader toolkit for selecting animal models best suited to address their specific biological questions.

## Introduction

Cnidarians (i.e., jellyfish, corals, sea anemones, and hydroids) represent a lineage that has been evolving independently from bilaterians for over 500 million years. This long period of independent evolution has enabled cnidarians to develop a distinct suite of characteristics, all governed by the same genetic toolkit shared with the common ancestor of Cnidaria and Bilateria. Cnidarians use a core conserved developmental toolkit to regulate processes such as axial patterning, cell type patterning, differentiation, and regeneration. Despite using similar genetic programs, the outcomes of body form, tissue organization, and organismal complexity are markedly distinct from the features observed within bilaterians. This deep genetic conservation, combined with their unique phylogenetic position, helps make cnidarians a key group for understanding the genetic basis of diverse body plans and biphasic life cycles. Moreover, comparative studies between cnidarians and bilaterians offer valuable insights into the origins and evolution of key traits, such as centralized nervous systems or the swimming medusa stage in jellyfish. The gain of novel traits like cnidocytes and specialized reproductive strategies in cnidarians also provides opportunities to probe how animals generate unique features from a core conserved gene tool kit and lineage-specific genes.

Cnidofest 2024 (https://www.cnidofest.org/) was held at Lehigh University, in Bethlehem Pennsylvania and drew attendees from 11 countries, promoting a diverse exchange of ideas and research perspectives. A total of 48 oral presentations were given based on selected abstracts, with 90% delivered by trainees, while the remaining 10% were presented by principal investigators. Following in the footsteps of Cnidofest 2018 [1] and 2022 [2], the 2024 meeting covered a broad range of topics and over 30 species of cnidarians were represented, with the largest number of talks focused on the long-standing laboratory models *Nematostella vectensis* and *Hydra vulgaris*. A feature of Cnidofest meetings is the technical talks, or “tech talks”, which were originally conceived at Hydroidfest, the predecessor of Cnidofest, to introduce cnidarian researchers to emerging and cutting-edge technologies. Cnidofest 2024 hosted three technology talks featuring Blair Benham-Pyle from Baylor College of Medicine, Yongxin Zhao from Carnegie Mellon, and Hang Lu from the Georgia Institute of Technology. The keynote address by Andrew Gordus from Johns Hopkins University discussed behavior and machine learning in orb-weaving spiders, providing an excellent example of building the tools and resources for a new model organism.

## Keynote: untangling the web of behaviors used in spider orb-weaving

The meeting kicked off with a Keynote address by Dr. Andrew Gordus, whose research focuses on understanding how neuronal circuits control both innate and learned behaviors, using *C. elegans* and orb-weaving spiders as model organisms. Neurobiology research has surged in recent years, particularly within invertebrate systems, as innovative studies on species like orb-weaving spiders are shedding light on the neural circuits that govern behaviors. Andrew’s innovative work in establishing the orb-weaving spider model system and developing methods to simultaneously quantify behavior and neural activity directly informs and serves as an inspiration to the growing field of cnidarian neuroscience, where similar technological adaptations are being made to study the development and function of nervous systems in species like *H. vulgaris, C. hemisphaerica*, and *N. vectensis,* and potentially additional species in the future.

Spider webs have long captured the attention of scientists and naturalists alike due to their remarkable complexity and repetitive geometric patterns, which often appear to follow an underlying mathematical logic. The process by which spiders construct their webs is a highly coordinated sequence of behaviors, unfolding in several distinct phases. Each phase is driven by specific actions that, when combined, result in the formation of a functional and intricate structure. The Gordus Lab has used advanced tracking techniques to study the behavior of orb-weaving spiders, revealing that web construction follows a series of predictable stages, with distinct leg movements occurring at each phase [3]. While these movements can vary from individual to individual, they exhibit a surprising level of consistency across spiders. Remarkably, the sequences of actions in each phase can be used to predict the final structure of the web, highlighting the connection between behavioral patterns and the physical architecture of the web. Gordus’ work on orb-weavers highlights how the physical structure of a spider web serves as a direct manifestation of the underlying behavioral processes and is part of a broader effort to understand how these behaviors are encoded in the spider’s brain.

## Technology talks

 A hallmark of the Cnidofest meetings have been the technology talks. One goal is to expose the cnidarian community to emerging tools while providing attendees the opportunity to engage with the pioneers and/or early adopters of the technology. This has translated to the community rapidly integrating emerging technology and an explosion of cnidarian research in recent years. For example, tech talks at previous meetings led to a rapid adoption of single cell omics and systems neuroscience approaches, which were on display throughout the 2024 iteration of Cnidofest. The tech talks for 2024 focused on expansion microscopy (Yongxin Zhao), spatial transcriptomics (Blair Benham-Pyle), and optogenetics (Hang Lu). These technologies were chosen to enable observations that will drive new hypotheses about the evolutionary origins of nervous system complexity, the functional roles of cnidarian-specific innovations (like cnidocytes), and the environmental factors that shape their diverse life cycles. As cnidarian research continues to integrate these powerful approaches, the field is poised to uncover fundamental principles of metazoan biology with implications far beyond this fascinating phylum.

### Using unbiased spatial transcriptomics to define cellular and molecular microenvironments

Our first technology speaker Blair Benham-Pyle (PI, Baylor College of Medicine) shared her expertise in single-cell mapping and spatial transcriptomics by discussing her work in the highly regenerative freshwater planarian *Schmidtea mediterranea*. Benham-Pyle’s approach combines trackable, single-cell RNA sequencing with fluorescence-activated cell sorting to interrogate cellular and molecular microenvironments. Leveraging single-cell mapping and spatial transcriptomics could help advance cnidarian regeneration research by revealing the molecular pathways and signaling networks that drive tissue repair and stem cell activation, as one example. These techniques would allow scientists to track how stem cells proliferate and differentiate over time, providing insights into the dynamics of regeneration. By examining the spatial organization of gene expression, researchers could better understand how cells coordinate to form functional tissues. Ultimately, this knowledge could inform regenerative medicine by identifying key mechanisms that could be applied to enhance tissue repair in other species, including vertebrates.

### Magnify as a molecular anchoring strategy for expansion microscopy

Yongxin (Leon) Zhao (PI, Carnegie Mellon) presented his groups recently developed ‘Magnify’ protocol for next-generation expansion microscopy. Expansion microscopy allows for high-resolution nanoimaging using standard microscopes by enlarging biological samples embedded in a crosslinked hydrogel. Early versions of expansion microscopy require reactive chemicals to anchor labels to the gel, but Zhao’s ‘Magnify’ technique uses a robust gel that retains nucleic acids, proteins, and lipids without this extra step. ‘Magnify’ can expand samples up to 11 times, achieving around 25-nm resolution with conventional microscopes and up to 15-nm resolution with super-resolution techniques, suitable for a range of specimens including brain synaptic proteins, kidney podocyte processes, and lung organoid structures [4]. This is particularly valuable for studying the complex tissue architectures of cnidarians, such as the intricate arrangement of neurons, cnidocytes, and epithelial cells, which could provide a new look into many aspects of cnidarian tissue biology. For example, morphogenetic movements during development or cell migration during regeneration events.

### Automating optogenetic manipulation for small invertebrates

Hang Lu (PI, Technology Talk, Georgia Institute of Technology) presented work that merges engineering with biology, focusing on microfluidic devices and biomedical (or biological) microelectromechanical systems to explore neuroscience, genetics, and biotechnology. Her group’s research spans the development and function of the nervous system, signal transduction in cancer therapy, and large-scale experimentation in systems biology, to name a few, ultimately aiming to develop new technologies for understanding and curing diseases. Animals use multiple mechanoreceptors to navigate their environments, and while previous studies on *Caenorhabditis elegans* have defined mechanosensory neuron roles, they often lack dynamic data. To overcome this challenge, Lu’s group employs a custom platform combining tracking, selective illumination, and optogenetics to analyze mechanosensory systems. Her approach provides a method to predict behavioral responses based on sensory neuron activity fitted for *C. elegans*, but is highly adaptable for a range of small invertebrates [5–7]. These lab-on-a-chip tools provide a unique perspective on biological systems, offering large-scale quantitative data through miniaturized devices that leverage micro and nano-scale phenomena. This microfluidic technology can be used in cnidarian research to analyze mechanosensory neuron activity and behavior in real-time, for example, enabling detailed investigations of neural circuit function and sensory processing in these organisms.

## Oral presentations

### Exploring current models and mechanisms in cnidarian regeneration

**Fig. 1:**
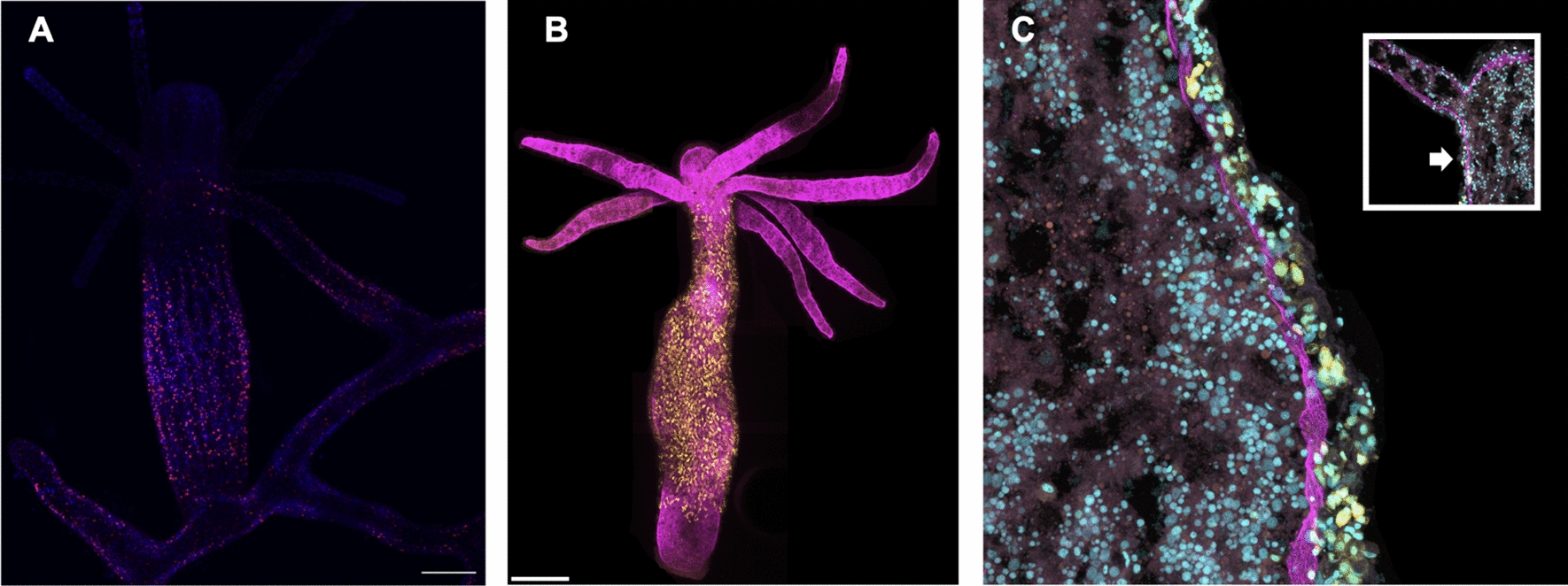
A strong cnidarian molecular toolkit. **A** Antibody staining against *Clytia* Yorkie (Yki) reveals protein localization in numerous *Hydractinia symbiolongicarpus* cells, with prominent expression in juvenile colonies and budding polyps. **B** Transgenic *H. vulgaris* expressing nanos:GFP shows GFP signal in yellow, with collagen I labeled in magenta. **C** Inset highlights the oral region of *H. vulgaris*, with the arrow indicating the magnified region shown in the main panel. Hoechst stains DNA in cyan. Scale bars: 250 μm (whole-animal views), 50 μm (magnified sections)

The conference featured a series of presentations on the mechanisms of tissue regeneration in cnidarians, highlighting the complexity and diversity of these regenerative processes. Ben Cox (postdoc, Juliano Lab, UC Davis) kicked the meeting off by discussing complex tissue remodeling during *H. vulgaris* head regeneration, which encompasses a suite of migratory and morphogenetic events and requires precisely coordinated regulation of the extracellular matrix (ECM). Cox found that by using a broad-spectrum matrix metalloprotease pharmacological inhibitor, normal ECM retraction was reduced during head regeneration, suggesting that matrix metalloproteinases (MMPs) are required for collagen degradation during head regeneration in *H. vulgaris.* Furthermore, Cox demonstrated using a nanos:GFP transgenic line and grafting experiment, that interstitial stem cells migrate from host tissue to invade and populate the grafted head of the regenerate (Fig. 1).

Figure 1 A strong cnidarian molecular toolkit. A Antibody staining against *Clytia* Yorkie (Yki) reveals protein localization in numerous *Hydractinia symbiolongicarpus* cells, with prominent expression in juvenile colonies and budding polyps. B Transgenic *H. vulgaris* expressing nanos:GFP shows GFP signal in yellow, with collagen I labeled in magenta. C Inset highlights the oral region of *H. vulgaris*, with the arrow indicating the magnified region shown in the main panel. Hoechst stains DNA in cyan. Scale bars: 250 μm (whole-animal views), 50 μm (magnified sections)

Marylene Bonvin (Graduate Student, Tsiairis Lab, Friedrich Miescher Institute for Biomedical Research) presented research investigating the role of metabolic signaling in cell fate decisions of *H. vulgaris* tissue patterning, utilizing transcriptomics and metabolomics. By overexpressing wnt, Bonvin observed a graded glycolytic activity along the body axis, implicating hexosamine pathways and O-glycosylation for efficient signaling during *H. vulgaris* tissue patterning. Iris Y Juanico (Graduate Student, Juliano Lab, UC Davis) continued by talking about the gene regulatory network (GRN) of regenerating *H. vulgaris*, focusing on connections between ERK signaling and the transcriptional activation of Wnt ligands by AP-1 transcription factors. By pharmacologically disrupting ERK signaling during head regeneration, Juanico discovered that *wnt9/10C* and *wntless* transcription is dependent on ERK signaling, but *wnt3* transcription is not. This reveals an unexpected complexity in the GRN driving regeneration. Clara Nuninger (Graduate Student, Tsiairis Lab, Friedrich Miescher Institute for Biomedical Research) continued by discussing the mechanism of morphallaxis in the regeneration of *H. vulgaris*, for which our current understanding is minimal. To better understand the spatial dynamics of regenerating axial identities, Nuninger performed a single-cell RNA sequencing experiment on regenerating *H. vulgaris* and found that cells at the regenerating extremities recreate their first missing neighbors and progressively begin acquiring their correct axial positions sequentially to adapt to a changing body size. Sera Weever (Graduate Student, Tsiairis Lab, Friedrich Miescher Institute for Biomedical Research) observed positive feedback between tissue stretching and Wnt3 signaling during *H. vulgaris* regeneration. Conclusions indicate that when cells experience greater stretching force, higher levels of Wnt3 are expressed. Additionally, cells that express higher levels of Wnt3 have a greater capacity for stretching. Supported by a mathematical model, which also does not require the presence of a long-range diffusible inhibitor. Moving on to another hydroid, Justin Waletich (Graduate Student, Schnitzler Lab, UF Whitney Marine Lab) identified eight new markers of the adult *Hydractinia symbiolongicarpus* pluripotent stem cell (i-cell) population from a single-cell atlas of the animal. In the atlas, one “germ-like” i-cell cluster and one “somatic-like” i-cell cluster were identified, five genes were expressed in both i-cell clusters (*Pcna*, *Nop58*, *Mcm4*, *Ubr7*, and *Uhrf1*) and three were expressed in one cluster or the other (*Zcwpw1 in the predicted germ i-cells; Pter and FoxQ2-like in the predicted somatic i-cells*). Analyses of spatial expression patterns showed that i-cell marker expression was dynamic and context dependent in the colony, though predicted somatic markers were exclusive to somatic tissues, and the predicted germ marker was exclusive to germ tissue. Differences were also seen in marker expression in regenerating polyp heads. At this time, no single marker has been identified that is exclusive to all i-cells. Yu-ichiro Nakajima (PI, University of Tokyo) presented on the regenerating tentacles of the hydrozoan jellyfish, *Cladonema pacificum.* Following tentacle amputation, Nakajima et al. found that during tentacle regeneration, repair-specific proliferative cells form a blastema in which cell proliferation is directly controlled by the ERK/MAPK signaling pathway, while proliferation of resident stem cells is controlled by Notch signaling independently of repair-specific programs following amputation. Together, these data suggest that appendage regeneration is managed by differential regulation of two distinct stem cell populations in *C. pacificum*.

## Exploring developmental innovation: gene dynamics and evolution, tissue remodeling, and cell type diversification

**Fig. 2:**
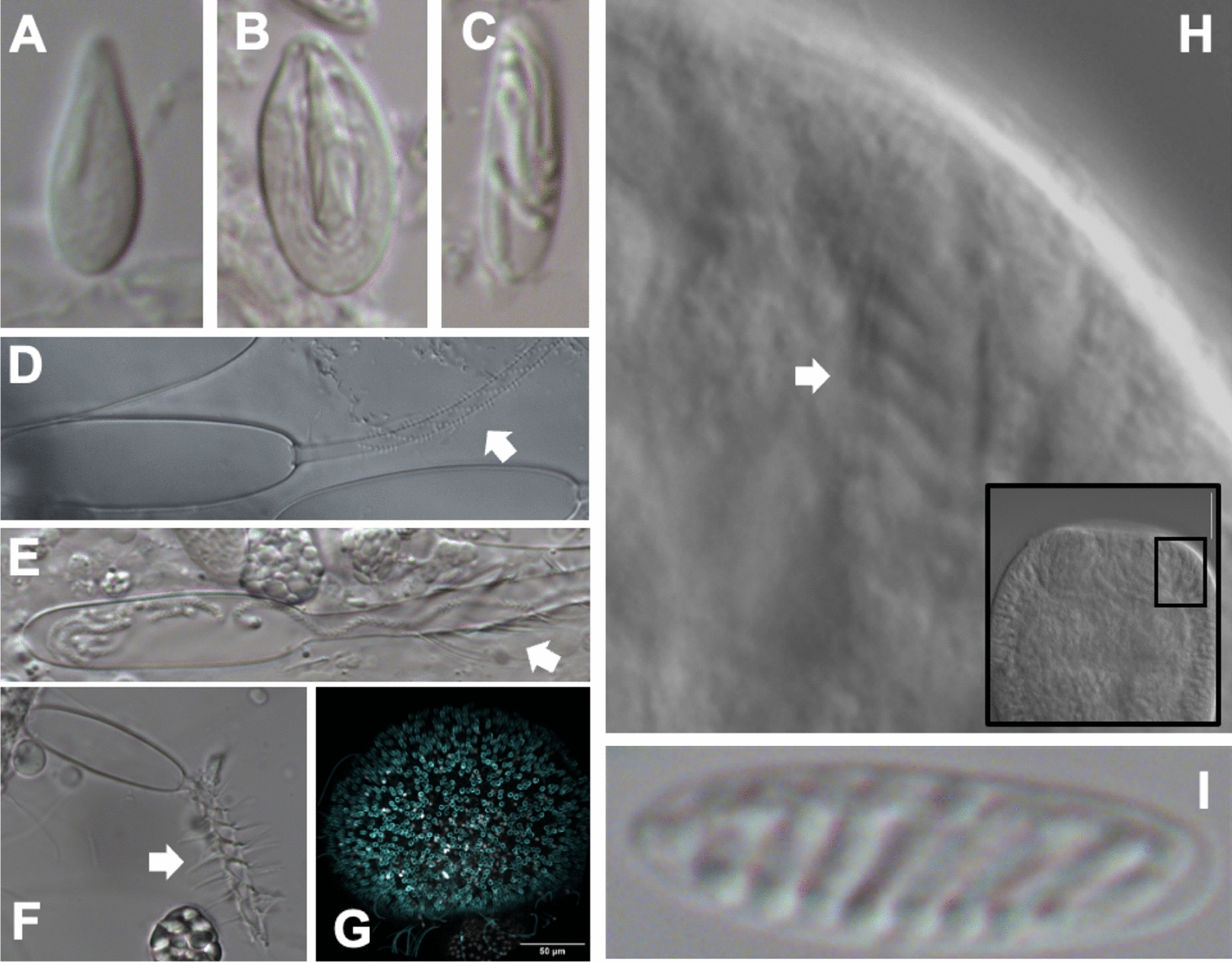
Cnidocyte diversity in two species. **A**–**C** DIC images of *Haliclystus sanjuanensis* cnidocytes. **D**–**F** DIC images of *Astrangia poculata* cnidocytes. White arrows highlight the heterogeneity of morphological features between each type. **G** mature *H. sanjuanensis* cnidocytes labelled with a minicollagen antibody. **H** DIC images of *A. poculata*, oral end (inset) and outset with white arrow showing large spirocytes. **I** Close up of *A. poculata* spirocyte under DIC

Kennedy Bolstad (Graduate Student, Babonis Lab, Cornell University) presented her work on tissue remodeling in the staurozoan, *Haliclystus sanjuanensis,* of which the adult life stage has adhesive structures called ‘anchors’ that are derived from remodeled juvenile tentacle tissue. A cell proliferation experiment confirmed a shift in investment from the juvenile tentacle to the adult anchor. Over the course of this transition, cnidocyte composition gradually becomes less dominated by eurytele and isorhiza types and instead comprised mainly birhopaloid types found in other parts of the body (Fig. 2).

Figure 2 Cnidocyte diversity in two species. A-C DIC images of *Haliclystus sanjuanensis* cnidocytes. D-F DIC images of *Astrangia poculata* cnidocytes. White arrows highlight the heterogeneity of morphological features between each type. G mature *H. sanjuanensis* cnidocytes labeled with a minicollagen antibody. H DIC images of *A. poculata*, oral end (inset) and outset with white arrow showing large spirocytes. I Close up of *A. poculata* spirocyte under DIC.

Lastly, Bolstad’s Edu experiments demonstrated that it takes 48 hours to replace fired cnidocytes in the feeding tentacles, and the next goal is to determine the timeline for anchor generation. Bailey Steinworth (Graduate Student, Martindale Lab, University of Florida) asked if in the scyphozoan *Cassiopea xamachana,* embryonic Hox genes are redeployed during asexual reproduction. Steinworth began by carefully describing polyp formation. First, bud formation begins from an outpocketing of the parent body wall, including epidermis, gastrodermis, muscle fibers, and symbionts. Then, gastrulation by invagination produces endoderm and ectoderm, giving rise to polyp gastrodermis and epidermis. In situ hybridization of Hox genes during this process revealed similar patterns in both the developing embryo and asexual bud, suggesting deployment of the same molecular toolkit in both reproductive strategies. Matthew Travert (Graduate Student, Cartwright Lab, Kansas University) shared his research on characterizing homeobox genes in medusozoans. He analyzed RNAseq data from four species: *Clytia hemisphaerica*, *Podocoryna carnea*, *Aurelia coerulea,* and *Craspedacusta sowerbii,* to identify genes specific to different life and stages and to assess stage-specific enrichment of homeobox genes. He identified 24 ANTP-class homeobox transcripts shared across all four species, with 33% showing medusa-specific expression (e.g., retinoic acid). Furthermore, ATAC-seq chromatin accessibility data supported the hypothesis that medusa development is regulated by retinoic acid [8]. Together, these data suggest that changes in the regulation of conserved metazoan genes might have played a role in the emergence of the medusa life stage. Sarah Arnold (Graduate Student, Babonis Lab, Cornell University) discussed pioneering the Northern Star Coral, *Astrangia poculata*, as a model for studying cell type diversification. Laboratory spawn induction has been achieved for this shallow water hard coral—inducible by heat shock—allowing larval manipulation. Arnold characterized the distribution of cnidocyte types in *A. poculata* embryos and adults, showing that both life stages possess a much broader diversity of cnidocytes than the equivalent stages in the sea anemone, *N. vectensis*. Interestingly, *A. poculata* larvae harbor a robust population of spirocytes (Fig. 2), like the mutant spirocyte found in *N. vectensis* Sox2 knockout animals [9].

## Recent advances in cnidarian genomics: unlocking genetic diversity

Namrata Ahuja (Graduate Student, Dunn Lab, Yale University) performed single-cell RNA sequencing on two siphonophore species (i.e., *Agalma elegans* and *Physalia physalis*) to identify cell types and better understand zooid specialization and development. Siphonophores are colonial cnidarians, recognizable by their pronounced sail (pneumatophore) and abundant tentacles. Throughout the clade, Ahuja identified cell clusters primarily marked with genes associated with the pneumatophore, cnidocytes, gland cells, and muscle cells, but did not find evidence for cell clustering according to zooid type (e.g., nectophore, gastrozooid, palpon). These data support the conclusion that different zooid types share developmental programs. Alberto Rivera (Postdoc, Baxevanis Lab, NIH) used genome annotation software (BRAKER) and protein structure prediction tools (AlphaFold2) to investigate the evolutionary history of allorecognition in cnidarians. His analysis of *P. carnea* allorecognition (Alr) genes revealed several candidate proteins that form a long, multimerized, contiguous complex. Notably, Rivera observed structural conservation between cnidarian Alr proteins and bilaterian immune system major histocompatibility complex proteins, suggesting an ancient evolutionary origin for this immune function. To further explore the evolutionary relationships of these proteins, Rivera compared Alr genes from *H. symbiolongicarpus* and *P. carnea*, finding evidence of lineage-specific gene duplications and syntenic conservation in the gene complex. These findings provide insights into the homology of Alr genes across species. Samuel Church (Postdoc, Dunn Lab, Yale University) presented work on the nearly globally distributed *P. physalis*. In combination with citizen science, Church obtained over 350 specimens from 14 countries and 133 specimens for whole genome sequencing. Clustering revealed support for at least four species and was further recapitulated in the >12,000 photographs obtained by private citizens via iNaturalist.com [10]. Together, the data support geographic separation of four separate species: Atlantic, Southern, IndoPacific/SW Atlantic and SW Pacific, and this determination loosely matches early morphological descriptions from the 1700 s and 1800s. In these sailing species, genetic variation is still highly partitioned geographically across the open ocean, restricting gene flow. Church further detected regionally endemic subpopulations, connected by winds and currents, and identified individual long-distance dispersal events. Darrin Schultz (Postdoc, Simakov Lab, University of Vienna) presented an analysis of 3,631 genomes from 2,291 species revealing that karyotype evolution primarily involves either contraction through chromosomal fusion and dispersion or expansion that disrupts ancestral linkage groups. Schultz also found that chromosomal changes correlate with extinction events and proposed that studying mixed state accumulation around key gene loci will be important for understanding clade-specific evolutionary innovations. He provided direct evidence for distinct macro-evolutionary trajectories of animal genome evolution by identifying topologically linked loci that migrate with important regulatory sequences, like the Hox cluster.

## A deep dive on cnidarian symbiosis: mechanisms, dynamics, and ecological impacts

Shumpei Maruyama (Postdoc, Cleves Lab, Carnegie Science) successfully isolated membrane-bound compartments containing the symbiotic *Symbiodiniaceae* spp. or ‘symbiosomes’ from the sea anemone *Exaiptasia diaphana.* Maruyama characterized the symbiosome proteome using tandem mass spectrometry and identified > 200 candidate proteins and proposed new hypotheses regarding the mechanisms underlying symbiosis formation, maintenance, and breakdown in *E. diaphana*. Miranda Gibson (Graduate Student, Titus Lab, University of Alabama) discussed using ddRADseq and ultra-conserved element phylogenomics to identify diversity in the tropical clownfish-hosting sea anemone *Radianthus magnifica*. Gibson sampled the magnificent sea anemone at six sites, including Moorea, Scattered Islands, Red Sea, Maldives, Japan, and Australia’s Great Barrier Reef. Principal component analysis of RADseq data revealed clustering for each region, with two distinct clusters at the Maldives site, likely due to the nature of the atoll’s geography. With an outer reef, inner lagoon and reef flat in between, Gibson obtained samples from each location and uncovered via discriminant PCA, a clear mating preference dividing reef flat anemones from inner lagoon and outer reef anemones. Sophie Macvittie (Graduate Student, Sogin Lab, University of California, Merced) used antibiotic cocktails to reduce bacterial load in the facultative symbiotic sea anemone *E. diaphana* and explored the impacts on host fitness. Macvittie found that after antibiotic exposure, sea anemone size and algal density were reduced, asexual reproduction was halted, and microbial load is reduced but begin recovery in one week - with full recovery after three weeks. Marker gene analysis during recovery showed a reduction of ASVs (amplicon sequencing variants, loosely representing individual bacterial species) but very little ASV difference during the recovery period. Further analysis of microbiome recovery relegated ASVs into four discrete bins: susceptible (e.g., *Rhodobacteraceae*), resistant (e.g., *Alteromonas*, *Halomona*), selected for (e.g., *Sphingomonadaceae, Pseudomonadaceae*), and opportunistic (e.g., *Alteromonadaceaea, Halomonadaceaea*).

## Expanding our understanding of cnidarian cell biology: development, differentiation, and regeneration

**Fig. 3:**
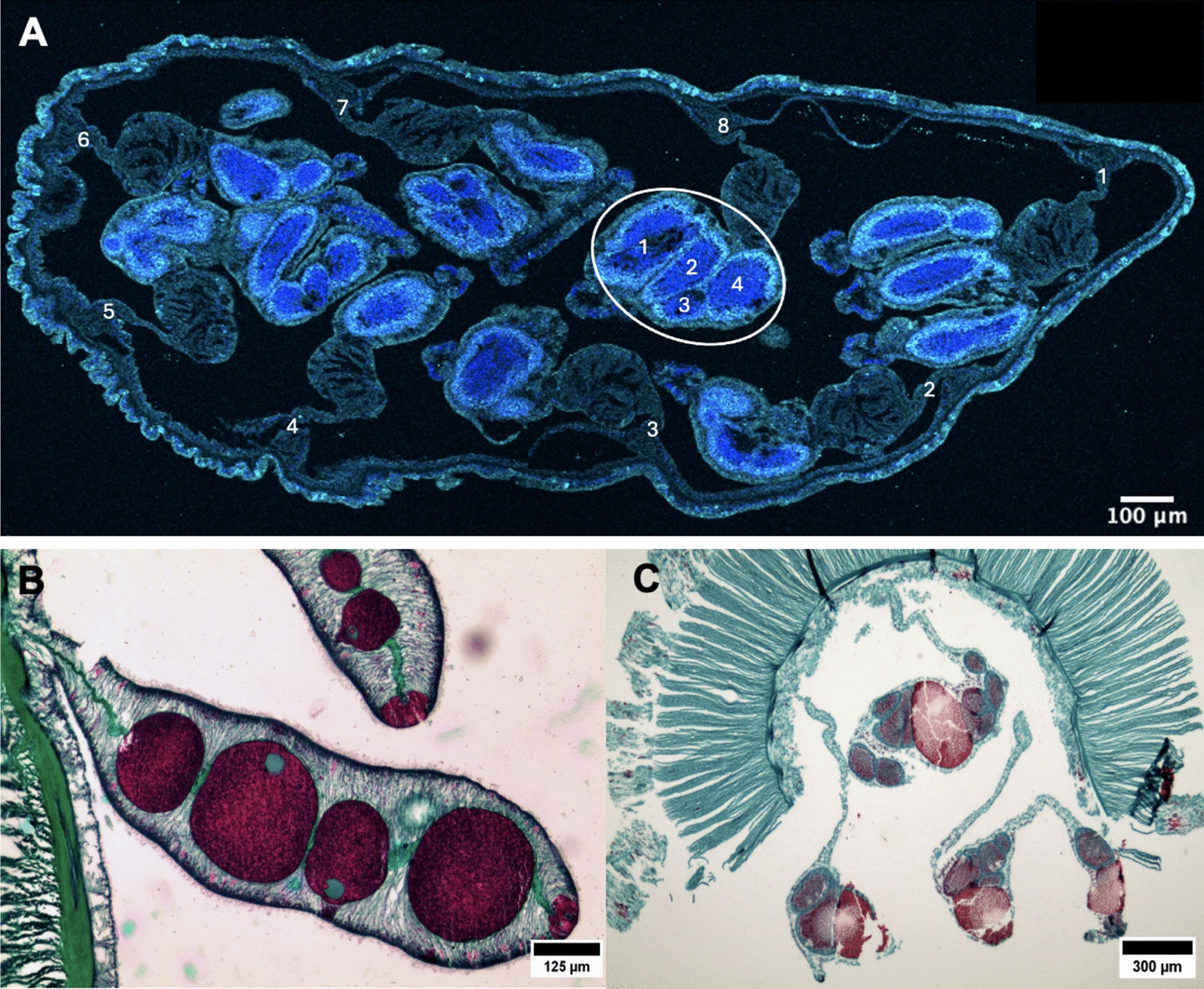
Reproductive traits in three cnidarian species. **A** *Nematostella vectensis* exhibits multiple large spermaries (circled region) per mesentery (numbered 1–8) suggesting a dioecious reproductive system. **B** *Cerianthus filiformis* contains large vitellogenic oocytes, likely a gonochoric species. **C** The presence of both large oocytes and spermaries within the same mesentery tissue in *Cerianthus vogti* indicates hermaphroditism

Sue Xu (Graduate Student, Schnitzler Lab, UF Whitney) discussed how the Hippo pathway regulates cell proliferation and tissue growth in *H. symbiolongicarpus*. Xu found, using in situ hybridization, that all hippo pathway genes are expressed in mature polyps, but only *hippo* and *yki* are expressed in budding polyps. Co-staining with an antibody against yki showed protein localization in many cells, especially in juvenile colonies and in budding polyps (Fig. 1). Further, pharmacological inhibition of *yki* with verteporfin decreases budding rates in juvenile colonies and head regeneration rates in feeding polyps, suggesting yki has a role in promoting budding and regeneration. David Ehrens (Graduate Student, Traylor-Knowles Lab, University of Miami) identified the decomposer commonly found in cnidarians’ mucus and tissues as thraustochytrids, a widespread oceanic protist. Ehrens proposed a protocol using Calcofluor White, a fluorescent blue dye that binds to cellulose and chitin, along with fluorescence-activated cell sorting (FACS) to identify and eliminate thraustochytrids from coral tissue samples, improving the accuracy of downstream analyses. Ines Fournon Berodia (Graduate Student, Steinmetz Lab, Michael Sars Center) discussed the cellular and molecular mechanisms underlying epithelial remodeling in response to food availability in the sea anemone *N. vectensis*. She posed the question of how an organism composed of only two cell layers maintains its structure during periods of starvation and cellular shrinkage. Through her research, she identified rosette-like epithelial structures, which are indicative of cell extrusion, and observed the activation of ERK1/2 (MAP kinase) signaling. These findings parallel similar mechanisms of cell extrusion observed in other organisms. Fournon Berodia proposed that *N. vectensis* is an ideal model for investigating the evolutionary origins of epithelial remodeling and for advancing studies on cell extrusion. Rong Xuan (Roy) Zang (Graduate Student, Musser Lab, Yale University) presented an examination of biogenic amine signaling, historically considered a bilaterian innovation, in the sponge *Spongilla lacustris.* Through targeted metabolomics, diverse biogenic amines were detected in several non-bilaterian species, including *S. lacustris*. In this sponge, tryptamine was shown to induce deflation by causing collapse of incurrent canals and expansion of excurrent canals. Two distinct cell types appear to mediate this process: metabolocytes, which secrete biogenic amines, and myopeptidocytes, which respond to them. Extracellular Ca2+ is necessary for incurrent canal contraction in sponges, while excurrent canal relaxation requires intracellular Ca2+. Stefanie Williams (Graduate Student, Gibson & Hawley Labs, Stowers Institute) analyzed the evolutionary origin of key molecular components of the synaptonemal complex in *N. vectensis* and characterized key meiotic steps with immunohistochemistry and microscopy. Williams determined that, in reproductively mature males, sperm are being produced and stored in ‘sperm bundles'within the gonads (Fig. 3) at high rates following acclimation to room temperature. This production continues even after spawning, until the animals are returned to cold conditions.

Figure 3 Reproductive traits in three cnidarian species. A *Nematostella vectensis* exhibits multiple large spermaries (circled region) per mesentery (numbered 1–8) suggesting a dioecious reproductive system. B *Cerianthus filiformis* contains large vitellogenic oocytes, likely a gonochoric species. C The presence of both large oocytes and spermaries within the same mesentery tissue in *Cerianthus vogti* indicates hermaphroditism

Michael Connelly (Postdoc, Baxevanis Lab, NIH) talked about his efforts to build a single-cell atlas for the hydrozoan *P. carnea* by leveraging the reference genome his group is currently drafting and annotating, combining long and short read data (i.e., illumina, PacBio HiFi, and Hi-C) to produce chromosome-scale scaffolds. The current assembly spans 616 megabases, organized into 34 scaffolds, with a scaffold N50 of 19.2 megabases. Using the BRAKER gene prediction pipeline with RNAseq data as supporting evidence, they generated over 38,000 gene models, capturing >90% of the metazoan single copy orthologs from the BUSCO dataset. Tim DuBuc (DuBuc Lab, CUNY Queens) presented work from his group assessing the effects of different pharmacological inhibitors of Wnt signaling in *H. symbiolongicarpus.* Three drugs, including a GSK3 inhibitor and an EZH2 inhibitor, elicited axis defects. Notably, the group identified a novel Wnt agonist that caused a striking loss of morphology and neuronal structures, as visualized using the *RFamide:GFP* transgenic line. When treating *Xenopus laevis* embryos with this inhibitor during early neurulation, a loss of eyes and disruption of normal development was observed, as well as a reduction in the number of RFamide:GFP neurons.

## Uncovering mechanisms of function, adaptation, and environmental response in cnidarian physiology

Daria Aleshkina (Graduate Student, Moran Lab, Hebrew University) talked about her work on the roles of RNA-dependent RNA polymerases (RdRPs) in the antiviral response of *N. vectensis*, a species with four RdRP homologs. When stimulated with poly(I:C), a synthetic analog of double stranded RNA (dsRNA) that mimics viral dsRNA, *N. vectensis* RdRP expression is upregulated. Differential expression analysis following knockdown of RdRPa1, RdRPa2, and RdRPb1 by siRNA revealed no developmental defects; however, CRISPR/Cas9 mutagenesis revealed that knocking out RdRPa1 leads to differential gene expression and loss of viral load regulation. Comparative genomics identified high conservation within *Hexacorallia*, especially among protein functional domains of the four RdRP homologs, suggesting that studies in *N. vectensis* will inform our understanding of viral response in corals. Janki Bhalodi (Graduate Student, Reitzel Lab, UNC Charlotte) used comparative genomics to identify the genome-wide distribution patterns of heat shock factor (HSF) binding sites in various cnidarians, including *N. vectensis*,* H. vulgaris*, *E. diaphana*, and *Acropora millepora.* Bhalodi identified as few as two and as many as five HSF genes in each cnidarian examined with some anthozoan specific genes identified. Benjamin Glass (Graduate Student, Barott Lab, University of Pennsylvania) discussed three species’ susceptibility to an experimentally simulated hypoxia event including larvae from *Galaxea fascicularis* and *Porites astreoides*, two tropical stony corals and *N. vectensis,* a brackish sea anemone*.* Results indicate a negative impact of hypoxia on early life stages for these species, including reduced swimming behavior and growth in all three, while zooxanthellae density and health, heat tolerance, and settlement in the obligately symbiotic coral species also suffered. Targeted metabolomics identified 81 metabolites across the three species impacted by hypoxia, many of which are implicated in the oxidation of fatty acids.

## Cutting-edge advances in cnidarian neurobiology: insights into neural circuits, behavior, and regenerative mechanisms

**Fig. 4:**
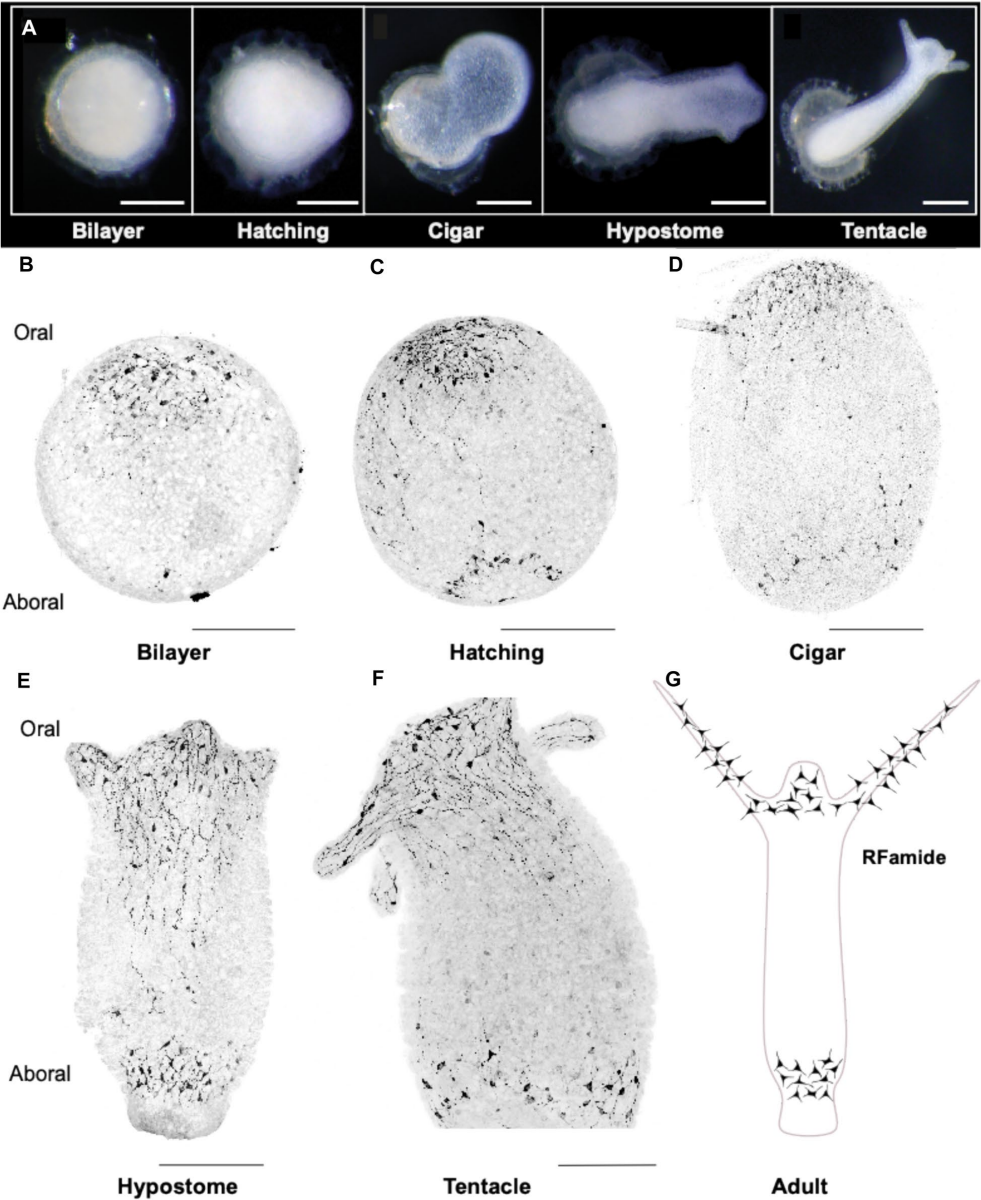
De-novo formation of neural networks in the developing *Hydra vulgaris* embryo. **A** Representative developmental stages of *H. vulgaris* embryos defined by key morphological transitions. Images were acquired using brightfield microscopy. Scale bars: 500 μm. **B**–**F** Immunolabelling of RFamide-expressing neurons across developmental stages: **B** Bilayer, **C** Hatching, **D** Cigar, **E** Hypostome, and **F** Tentacle formation stages, respectively. **G** Schematic representation of RFamide neuropeptide expression in the adult *H. vulgaris* nervous system

Wataru Yamamoto (Postdoc, Yuste Lab, Columbia University) is using the freshwater species, *H. vulgaris,* to explore neural mechanisms driving osmoregulation. After water accumulates in *Hydra*’s gut cavity, it is expelled through the mouth, but the mechanisms governing this action remain unclear. Yamamoto shed light on this behavior by quantifying water excretion and found that upon expulsion, *H. vulgaris* experiences a 15% reduction in body size within 5–10 s, approximately every 2 hours, but not under high osmolarity conditions. By expressing a fluorescent calcium indicator, they found that RP2 neurons were active just before water excretion and became inactive immediately afterward. Further, two-photon ablation of RP2 neurons decreased spontaneous contractions and body length. Ehsan Sakib (Research Assistant, Christian Albrechts University, Bosch Lab) is using asexual development in *H. vulgaris* to understand how a new nerve net develops and ultimately gains independence from the parent polyp. Using a combination of cell ablation, GCaMP6s imaging, and immunohistochemistry, the study revealed that sequential differentiation of specific neuronal populations, En1and Ec4B, is the primary driver of dense nerve net formation at the oral end. This enables an independent mouth opening in the developing bud. Christopher Noack (Graduate Student, Christian Albrechts University, Bosch Lab) developed a methodology to investigate the formation of neural networks de novo in the developing *H. vulgaris* embryo. Using a combination of immunohistochemistry and high-resolution imaging techniques (Fig. 4), he was able to examine the synchronization of specific neuronal populations during critical stages of late embryogenesis. This enabled observation of how neurons progressively develop coordinated activity, a fundamental step in the formation of a functional neural network.

Figure 4 De novo formation of neural networks in the developing *Hydra vulgaris* embryo. A Representative developmental stages of *H. vulgaris* embryos defined by key morphological transitions. Images were acquired using bright-field microscopy. Scale bars: 500 μm. B–F Immunolabelling of RFamide-expressing neurons across developmental stages: B bilayer, C hatching, D cigar, E hypostome, and F tentacle formation stages, respectively. G Schematic representation of RFamide neuropeptide expression in the adult *H. vulgaris* nervous system

Hannah Justin (Graduate Student, UNC Charlotte, Reitzel Lab) constructed an innovative platform designed to automate the delivery of mechanical stimuli to *N. vectensis*, with the aim of assessing the organism's capacity to habituate to repeated stimuli. This system allows for the precise and consistent application of non-lethal mechanical forces, delivered in a controlled and repeated manner. The resulting behavioral responses of the sea anemones were then analyzed using the advanced machine learning software SLEAP, which enables the identification and quantification of subtle behavioral patterns over time and offers valuable insights into the mechanisms underlying learning and memory in cnidarians. Marc Meynadier (Graduate Student, Laboratoire de Biologie du Développement de Villefranche, Copley Lab) presented results showing the mapping of equivalent cell types across cnidarian species, consistent with manual annotations and benchmarked tools. Functional annotation reveals significant overlap between homologous genes of cnidocytes and neurons, providing insights into their common ancestral state and cell type evolution. The study highlights how single-cell data can reveal shared genetic features across species and provide insights into broad evolutionary trends. Julia Baranyk (Graduate Student, Nakanishi Lab, University of Arkansas) reported on evidence for cryptic diversity of mechanosensory neurons in the ectodermal epithelium of *N. vectensis* feeding tentacles. Two morphologically distinct presumptive mechanosensory neurons, conventional type I hair cells and previously unrecognized type II hair cells, differ in apical sensory apparatus structure and synaptic connectivity patterns. Functional experiments suggested the newly identified mechanosensory neuron type allows animals to respond to prey in close proximity using long sensory cilia, to better position tentacles for prey capture. Julia Ramon-Mateu (Graduate Student, Laboratoire de Biologie du Développement de Villefranche, Copley Lab) conducted an analysis to identify genes that are preferentially expressed in the aboral versus oral halves of planula larvae, utilizing both RNA-seq and scRNA-seq techniques. Ramon-Mateu used planula larvae from three distinct cnidarian species representing the two major clades within the phylum: the medusozoan jellyfish *C. hemisphaerica* and the anthozoan stony corals *Astroides calycularis* and *Pocillopora acuta*. By combining these transcriptomic approaches, she was able to generate detailed gene expression profiles that highlighted regional differences in the planulae, providing valuable insights into the molecular mechanisms underlying early developmental patterning.

## Exploring cnidarian development: from neurogenesis to regenerative mechanisms

MingHe Cheng (Graduate Student, Layden Lab, Lehigh University) presented an investigation of neuronal subtype identity patterning in *N. vectensis*. By using scRNA sequencing combined with colorimetric and fluorescent in situ hybridization to track expression of achaete-scute homolog, *NvashA*, Cheng found support for at least two waves of neurogenesis. These approaches allowed for a high-resolution analysis of gene expression patterns across individual cells, providing a comprehensive view of the neurogenic landscape. Through these methods, Cheng was able to predict and then directly observe the emergence of distinct neural clusters at the late planula stage. These neural clusters, which were identified based on their unique gene expression profiles, suggest that the developing nervous system in *N. vectensis* undergoes substantial diversification at this stage, likely corresponding to different neuronal subtypes that will later participate in various functional roles within the organism. Benjamin Danladi (Graduate Student, Layden Lab, Lehigh University) presented his work on the role of the transcription factor *NvfoxD-like2* in *N. vectensis* cnidogenesis. He showed that at least three regulatory pathways govern cnidocyte formation in *N. vectensis*, including core pan-cnidarian pathways and a lineage-specific pathway driven by *NvfoxD-like2*, which may contribute to hexacorallian or *N. vectensis*-specific cnidocyte characteristics. Danladi used shRNA knockdown, scRNA-seq, and bulk RNA-seq to determine that *NvfoxD-like2* is expressed in cnidocytes at all stages of early development but does not appear to regulate known cnidocyte pathway genes (e.g., *NvSoxC*, *NvSoxB(2)*, or *Nvznf845*). Bert Hobmayer (PI, University of Innsbruck) presented work on the function of *Myc* in *H. vulgaris*. By performing siRNA knockdown of *Myc2* followed by bulk RNA sequencing, Hobmayer and Lechable observed downregulation of several known pluripotency factor-encoding genes such as *piwi*, *vasa*, *argonaute*, *tudor*, *mago nashi*, etc., as well as a block of ribosome biosynthesis. Importantly, they observed inhibition of cell cycle progression in interstitial stem cells, and the upregulation of genes involved in nematocyte differentiation. Taken together, these data suggest that *Myc2* acts as a stem cell maintenance factor in *H. vulgaris* and supports an ancestral role for this gene as a master regulator of the cell cycle. Hannah Morris Little (Graduate Student, Juliano Lab, University of California at Davis) presented her work on Wnt signaling in *H. vulgaris*. Morris Little hypothesized that Wnt signaling directs the expression of different target genes in distinct cell types, such as the ectodermal and endodermal cells, acting in concert with cell type-specific transcription factors. To test this, she is leveraging positional and cell-type information from a single nuclei multi-omics (paired snATAC-seq + snRNA-seq) dataset. This approach allows her to map the range of Wnt signaling responsiveness along the oral–aboral axis and identify differentially accessible chromatin regions across cell types. Notably, putative Wnt target genes exhibit accessible cis-regulatory elements that are cell type specific, supporting the idea that Wnt signaling regulates distinct transcriptional programs depending on cellular context. These findings represent an important step toward defining how Wnt signaling shapes cell type-specific gene regulatory networks in *H. vulgaris*. Ulrich Technau (PI, University of Vienna) presented recent work from his group showing that together with Wnt and MAPK signaling, Notch signaling regulates ‘endodermal’ (i.e., pharyngeal, ectodermal) specification in *N. vectensis*, which may be a shared ancestral feature with bilaterians. Regeneration experiments showed that during polyp regeneration, Notch is necessary to establish germ layer identities. Together, these data support an ancestral role for Notch signaling in axis formation and germ layer segregation in the sea anemone *N. vectensis*. Layla Al-Shaer (Postdoc, Layden Lab, Lehigh University) has produced one of the first differential expression (DE) analyses of both male and female *N. vectensis* peri-induction of their spawning cycles. DE results revealed vast differences in the transcriptional profiles of each sex, but by mapping those transcripts to available scRNAseq atlases Al-Shaer showed that the ‘reproduction program’ is likely a whole-body response and not isolated to a one or a few cell types. A comparative analysis with *A. millepora* RNAseq data from Kaniewska et al. (2015) indicates there may be conservation of the reproductive mechanisms in anthozoan cnidarians including differential expression of neuropeptides, opsins and other GPCRs. Mako Takahashi (Graduate Student, Kumano Lab, Tohoku University) investigated the temporal landscape of female germline cell determination in *C. pacificum*, where oocytes begin development in the manubrium 4–6 days after formation of the first tentacle branch. In situ hybridization showed that *Nanos2* was expressed both in putative i-cells of the manubrium and in growing oocytes, while *Nanos1*, *Vasa1*, *Vasa2* and *Piwi* were expressed only in the oocytes. In contrast, i-cells that accumulate in the tentacle bud express *Nanos1*, *Vasa1*, *Vasa2* and *Piwi*, but not *Nanos2*, suggesting that i-cells of the manubrium and those of tentacle buds are transcriptionally distinct. Sergio Stampar (PI, São Paulo State University) conducted ultrastructural analysis of reproductive tissues from 18 *Ceriantharia* (tube anemones) species across eight genera. Stampar’s findings reveal diverse reproductive strategies (Fig. 3), including gonochorism, sequential hermaphroditism, and gynodioecy, characterized by populations composed of females and hermaphrodites but lacking male-only individuals. Yamini Ravichandran (Postdoc, Roux Lab, Université de Genève) concluded the meeting with a presentation on the function of topological defects in actin organization during head regeneration in *H. vulgaris*. *H. vulgaris* tissues display properties reminiscent of liquid crystals, and Ravichandran presented evidence that topological defects in the actin cytoskeleton participate in initiating regeneration from small tissue fragments. By applying pressure with an agar slab to induce these defects, she observed a strong correlation between defect formation and successful head regeneration [11]. In contrast, when the tissue was compressed to form a toroid or “donut” lacking topological defects, regeneration failed to occur. These findings are reinforced by the presence of similar topological defects in other biological systems, such as in vitro cell monolayers, cell extrusion, and cell sorting. Together, these results support the idea that actin-based topological defects are a conserved mechanism contributing to morphogenesis across diverse animal systems.

## Posters

A total of 37 posters were presented across two poster sessions. All presenters were given the option to give a 2-min lightning talk prior to the session to garner attention for their posters. Organizers and attendees agree this is one of the most fun aspects of Cnidofest. Three categories of awards based on career stage were presented with prizes to 1 st and 2nd place winners. Two undergraduate awards were presented at the meeting. The first was granted to Jackson Crane, a postbaccalaureate researcher in the Juliano Lab, for his presentation on the mechanisms of Wnt signaling during early foot regeneration in *H. vulgaris*. The second award went to Atharva Valanju, a visiting masters research student hosted in the Gibson lab, who discussed the molecular tools regulating axial segmentation during the development of *N. vectensis*. Additionally, two graduate student awards were presented: Kent Winata from the Cartwright Lab at the University of Kansas, for his transcriptomic analysis and differential gene expression between life cycle stages of the freshwater jellyfish *Craspedacusta sowerbii*, and Valeria Dountcheva from the Rouhana Lab at the University of Massachusetts-Boston, for her work on maternal mRNA regulation by cytoplasmic polyadenylation across the animal kingdom. Finally, in the postdoctoral category, three researchers were honored with awards: Zac Lane from the Schnitzler Lab at University of Florida Whitney Marine Lab, for his development of flow cytometry methodologies in the colonial hydroid *H. symbiolongicarpus*; Christophe Dupre from the Engert Lab at Harvard University for his work on cold acclimation as a robust overwintering strategy in *H. vulgaris*; and Jamie Havrilak from the Layden Lab at Lehigh University, for utilizing single-cell RNA sequencing to identify neural subtypes in *N. vectensis*.

## Summary

Cnidofest 2024 was a resounding success, showcasing the rapid advancements in cnidarian neurobiology and the growing integration of organismal biology, neuroscience, and behavior. This year’s meeting highlighted how cutting-edge approaches link specific neurons to behaviors in cnidarians—a breakthrough achieved in *H. vulgaris* [12–15] and now within reach for several other cnidarian species. A standout aspect of the meeting was the exceptional quality of presentations, particularly from early-career researchers, demonstrating both the field’s momentum and its promising future.

This year’s technological highlights included spatial transcriptomics, optogenetic control of ion channels, and expansion microscopy. Spatial transcriptomics enables the mapping of gene expression across tissues while maintaining cellular organization. Optogenetics and chemogenetics can provide real-time manipulation of neural activity to study cnidarian behavior, sensory processing, and regeneration. When paired with high-throughput screening and machine learning, this technology will accelerate the testing of genetic, chemical, and environmental factors that impact nervous system function in cnidarians. Their small body size makes many cnidarian species ideal for whole nervous system imaging in live, behaving animals. Thus, this group of animals has significant potential to impact our fundamental understanding of neurobiology.

Expansion microscopy is further transforming the field by providing a cost-effective and accessible way to visualize subcellular structures without the need for electron microscopy. This approach is particularly well suited to cnidarians, where traditional ultrastructural studies have been limited. By expanding tissues for high-resolution light microscopy, researchers can explore unique features like bidirectional synapses and cnidocyte architecture in new detail, as well as uncover previously unappreciated aspects of cnidarian cell biology. Cnidarians continue to offer unique insights into nervous system evolution, regeneration, and plasticity, while also informing broader questions in developmental, cell, and evolutionary biology. As tools for probing cell types, regulatory mechanisms, and neural function in these animals improve, we anticipate transformative discoveries that will impact not only cnidarian research but also deepen our understanding of fundamental biological processes across metazoans. The momentum from this year’s meeting sets the stage for an exciting future, and we look forward to seeing how far the community will progress by the next meeting, scheduled for September 2026 at the Marine Biological Laboratory in Woods Hole, MA.

## Data Availability

No datasets were generated or analysed during the current study.
